# Association between class III obesity and overall survival in previously untreated younger patients with acute myeloid leukemia enrolled on SWOG S1203

**DOI:** 10.21203/rs.3.rs-4020184/v1

**Published:** 2024-03-11

**Authors:** Mary-Elizabeth Percival, Michelle Zhang, Megan Othus, Kerry McMillen, Harry Erba, Guillermo Garcia-Manero, John Pagel, Mohamed Sorror

**Affiliations:** University of Washington; University of Washington; Fred Hutchinson Cancer Research Center; Fred Hutchinson Cancer Center; Duke University; The University of Texas M.D. Anderson Cancer Center; Swedish Cancer Institute; Fred Hutchinson Cancer Research Center

## Abstract

There has been ongoing debate on the association between obesity and outcomes in acute myeloid leukemia (AML). Currently there are few studies that have stratified outcomes by class I obesity, class II obesity, and class III obesity; and a more nuanced understanding is becoming increasingly important with the rising prevalence of obesity. We examined the association between body mass index (BMI) and outcomes in previously untreated AML in younger patients (age ≤60) enrolled in SWOG S1203 (n=729). Class III obesity was associated with an increased rate of early death (p=0.004) and worse overall survival (OS) in multivariate analysis (hazard ratio (HR) 2.48, 95% confidence interval (CI) 1.62–3.80 versus normal weight). Class III obesity was also associated with worse OS after allogeneic hematopoietic cell transplant (HR 2.37, 95% CI 1.24–4.54 versus normal weight). These findings highlight the unique risk of class III obesity in AML, and the importance of further investigation to better characterize this patient population.

## Introduction

The prevalence of obesity is rapidly increasing in the United States, rising from 31% to 42% from 1999 to 2017.^1^ This is reflected in the rates of obesity in patients with AML, which increased from 36% in 1997 to 49% in 2010.^2^ Obesity is associated with increased risk of solid organ and hematologic malignancies, including myelodysplastic syndrome (MDS) and acute myeloid leukemia (AML).^3,4^ While obesity has been correlated with increased mortality in the general population, the association between obesity and outcomes in patients with newly diagnosed acute myeloid leukemia (AML) has been inconsistent.^2,5–9^ Given the increasing prevalence of obesity, it is increasingly important to understand the association between obesity and outcomes in patients with AML.

Studies looking at the impact of obesity in patients with AML commonly define obesity as body mass index (BMI) ≥ 30 kg/m^2^. Obesity can be further subdivided into class I obesity (BMI 30 to <35 kg/m^2^), class II obesity (BMI 35 to <40 kg/m^2^) and class III obesity (BMI ≥40 kg/m^2^). Accumulated evidence indicates that class II and III obesity are associated with increased health risks.^10–12^ A recent study found that class I obesity is associated with a 25% increase in health care expenditure compared with normal BMI, while class III obesity is associated with a 100% increase. ^13^ We set out to examine the association between classes of obesity and outcomes in patients with AML treated with induction chemotherapy and allogeneic hematopoietic cell transplant (HCT). Further stratification of at-risk patient populations could aid in design of future interventions.

## Methods

### Study Population

We retrospectively reviewed a group of 738 patients with previously untreated AML, as defined by World Health Organization (WHO) criteria, as part of clinical trial SWOG S1203. This trial was conducted by the SWOG Cancer Research Network that enrolled patients between 2012 and 2015.^14^ Inclusion criteria were age 18–60 years old, no severe comorbidities precluding intensive chemotherapy, and normal cardiac function. SWOG criteria previously described by Guillermo et al. were used to classify cytogenetic risk. Nine patients who did not receive protocol therapy or had missing BMI were excluded from analysis, leaving a final study analysis cohort of 729 patients.

Subjects were randomized to one of three induction chemotherapy arms: daunorubicin and cytarabine (DA), idarubicin and cytarabine (IA), and IA with vorinostat. Treatment for the DA arm included: cytarabine 100 mg/m^2^/day intravenous (IV) on days 1–7 and daunorubicin 90 mg/m^2^/day IV on days 1–3. Treatment for the IA arm included: cytarabine 1500 mg/m^2^/day IV on days 1–4 and idarubicin IV 12 mg/m^2^/day days 1–3. Treatment for the IA and vorinostat arm included: oral vorinostat 500 mg three times per day on days 1–3, cytarabine 1500 mg/m^2^/day IV on days 4–7 and idarubicin 12 mg/m^2^/day IV days 4–6. Dosing was based on body surface area (BSA) for cytarabine, but a BSA cap of 2.5 m^2^ was used for daunorubicin and idarubicin. Subjects with adverse risk cytogenetics were recommended to receive HCT during first complete remission (CR1).^14^

We used body mass index (BMI) cutoffs per the WHO to define underweight (BMI <18.5 kg/m^2^), normal weight (BMI 18.5 to <25 kg/m^2^), overweight (BMI 25 to <30 kg/m^2^), class I obesity (30 to <35 kg/m^2^), class II obesity (35 to <40 kg/m^2^), and class III obesity (BMI≥40 kg/m^2^). Weight was collected on first day of induction chemotherapy. Early death was defined as death from any cause within 28 days of induction chemotherapy. Complete remission (CR) was defined as ANC ≥ 1,000/mcl, platelet count 100,000/ mcl, no Auer rods, <5% bone marrow blasts, and no evidence of extramedullary disease. Complete remission with incomplete count recovery (CRi) needed to meet criteria of CR except that ANC may be < 1,000/mcl or platelet count < 100,000/mcl. Other outcome measures included overall survival and overall survival after transplant.

### Statistical Methods

Overall survival (OS) was measured from date of randomization to date of death from any cause with patients last known to be alive censored at date of last contact. Multivariable time-dependent Cox regression models were used to evaluate covariate (quantitative unless otherwise specified) associations between weight category (referenced to normal weight), age at randomization, sex (male versus female), race (white versus not white), Eastern Cooperative Oncology Group (ECOG) performance status (2–3 versus 0–1), secondary disease (versus de novo), WBC, platelets, marrow blasts, cytogenetic risk (intermediate, adverse or unknown versus favorable), FLT3 (TKD or ITD versus wildtype) with OS. HCT in first complete remission (CR1) was included as a time-dependent covariate. Fisher’s exact test and Wilcox rank sum tests were used to compare covariates across randomized arm. Two-sided tests are reported and a p-value <0.05 was considered statistically significant.

## Results

Median age was 49 years (range, 18 to 60), 51% were male, and 88% had a performance status (PS) of ECOG 0–1 (Table 1). The majority were white (83%). Most patients had de novo disease (90%) and intermediate cytogenetic risk (63%). The range of different BMI categories was broad: only 1% were underweight, 25% were normal weight, 31% were overweight, 20% had class I obesity, 11% had class II obesity and 12% had class III obesity. Increased BMI correlated with increased rates of poor performance status (ECOG 2–3: underweight 0%, normal weight 7%, overweight 12%, class I obesity 12%, class II obesity 13%, class III obesity 26%; p = 0.002).

There was no evidence of a difference in the rate of achieving composite CR (CR with or without count recovery) between different weight categories (p = 0.18). When early death rate was evaluated among all patient subgroups, no clear trend was identified (underweight: 0%; normal weight: 2%; overweight: 4%; class I obesity: 5%; class II obesity: 1%; class III obesity: 10%; p = 0.05). However, the rate of early death was significantly higher in the class III obesity group (10%) compared to all the other groups combined (3%; p = 0.004) and compared to normal weight (2%; p = 0.006).

**Figure 1:**
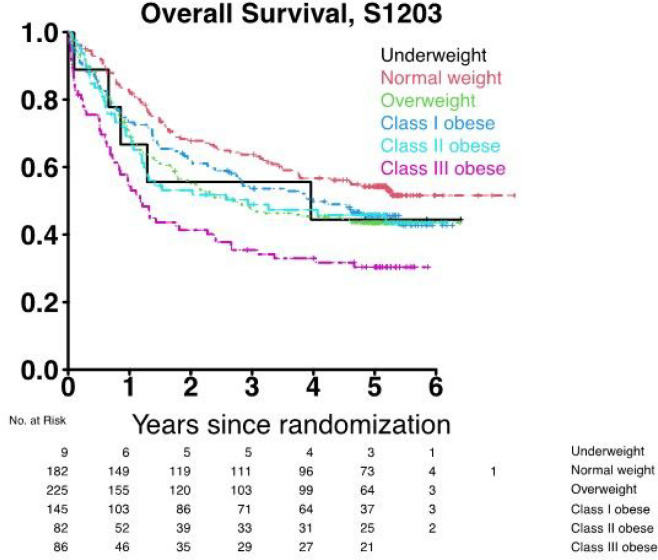
Overall survival stratified by BMI

Further, class III obesity was associated with lower OS: 3-year OS = 36%, 95% confidence interval (CI) 27–47% as compared to underweight (56%, 95% CI 31–100%) normal weight (64%, 95% CI 57–71%), overweight (47%, 95% CI 41–54%), class I obesity (54%, 95% CI 46–63%) and class II obesity (49%, 95% CI 39–62%) (Fig. 1). In multivariable analyses, class III obesity was significantly associated with worse OS (hazard ratio (HR) 2.48, 95% CI 1.62–3.80 versus normal weight). While OS was worse among subjects with class I (HR 1.37, 95% CI 0.93–2.03) and class II obesity (HR 1.46, 95% CI 0.94–2.29) compared to those with normal weight, these associations did not reach statistical significance (Fig. 1). Other factors that remained significantly associated with OS after multivariable adjustment included age (HR 1.03, 95% CI 1.01–1.04) and cytogenetic risk (intermediate: HR 2.44, 95% CI 1.32–4.50; adverse: HR 5.86, CI 3.10–11.06 versus favorable). HCT during CR1 was not found to be significantly associated with OS (HR 0.88, CI 0.67–1.15).

In multivariable cause-specific regression model for time to transplant in CR1, no significant association was identified between any BMI group and time to HCT. Intermediate (HR 4.03, 95% CI 1.73–9.38 versus favorable) and adverse risk disease (HR 7.14, 95% CI 2.98–17.11 versus favorable), FLT3-ITD (HR 2.80, 95% CI 1.90–4.11), and secondary disease (HR 1.66, 95% CI 1.06–2.58) were associated with increased time to transplant whereas presence of an *NPM1* mutation (HR 0.62, 95% CI 0.41–0.95) was associated with decreased time. Multivariable regression analyses of OS after HCT during CR1 demonstrate that class III obesity (HR 2.37, 95% CI 1.24–4.54 versus normal weight) and older age (HR 1.02, 95% CI 1.00–1.04) were significantly associated with worse OS. Class I obesity (HR 1.33, 95% CI 0.78–2.25 versus normal weight) and class II obesity (HR 1.76, 95% CI 0.97–3.17 versus normal weight) were not associated with worse OS.

## Discussion

There has been mixed evidence on the association between obesity and outcomes in patients with AML. ^2,6–8,15^ Here, we made use of a uniform prospectively enrolled group of newly diagnosed younger patients with AML in SWOG trial 1203, where we could show class III obesity was associated with decreased OS both overall and after HCT in CR1. Our results suggest that the degree of obesity may be negatively correlated with outcomes. The pathophysiology of the effect is unknown, but notably while class III obesity was associated with increased early death rate, it was not associated with rate of composite CR. Similarly, for the subset who received HCT, class III obesity was correlated with decreased OS after HCT after adjusting for covariates.

This study evaluated the findings in one clinical trial in adult patients age 60 or younger that excluded patients with cardiac co-morbidities such as heart failure, new or unstable angina, and prolonged QTc. Class III obesity is known to be independently associated with increased mortality from heart disease, cancer, and diabetes, and overall reduction in life expectancy.^16,17^ No comorbidity data were available, and it would have been interesting to examine if comorbidities, such as diabetes, were associated with infection risk or other complications. In addition to comorbidity burden, there have been other proposed mechanisms for worse outcomes in class III versus class I obesity. The “obesity paradox” has been identified in cardiovascular disease, where patients with class I obesity have a more favorable prognosis compared to individuals who are normal or underweight. Some patients with BMI ≥ 30 kg/m^2^ may have an athletic build and thus increased lean mass.^18^ Sarcopenic obesity, on the other hand, has been associated with worse prognosis and functional capacity in heart failure. ^19,20^ Future studies investigating the intersection of sarcopenia and obesity in patients with AML may help identify opportunities for intervention to improve outcomes.

Chemotherapy dosing strategy may also impact outcomes in obese patients, and patients in the S1203 study had a BSA cap for anthracycline dose. This study preceded the 2021 American Society of Clinical Oncology (ASCO) guideline recommending full weight-based cytotoxic chemotherapy in obese adults with cancer.^21^ Weight based chemotherapy dosing is generally not associated with increased toxicity in obese patients, and under-dosing can be associated with worse outcomes. Other studies indicate that different dosage strategies for AML (including total body weight, dosage capped, idealized body weight, and adjusted body weight) were not associated with changes in OS, CR or increased chemotoxicity. ^22,23^ Many AML patients ultimately undergo allogeneic HCT, and a study by Doney *et al*. found that patients with BMI ≥ 35.0 kg/m^2^ had worse non-relapse mortality associated with increased conditioning intensity.^24^

There have also been mixed findings on the association between obesity and outcomes after HCT in patients with myeloid malignancies.^25–27^ A multicenter study of 4215 patients with AML did not find any differences in transplant-related mortality or OS in obese patients (BMI > 30 to 34 kg/m^2^) and morbidly obese patients (BMI ≥ 35 kg/m^2^). ^28^ In contrast, another study of patients with AML undergoing allogeneic HCT found that obese patients (BMI ≥ 30 kg/m^2^) had higher rates of non-relapse mortality and shorter OS after HCT.^29^ The subjects included in our analyses were relatively homogenous, which may have reduced confounding factors present in prior studies. Our study also analyzed class III obesity (BMI ≥ 40 kg/m^2^) separately, and found that Class III obesity seemed to portend worse outcomes than class I and II obesity after HCT. It is unlikely that delayed transplantation in obese patients in our analyses contributed to the worse outcomes, since there was no significant difference in the time to transplant between different BMI groups. Interestingly, receipt of HCT was not associated with increased OS in multivariate analysis. This could be due to the study design: all adverse risk patients were recommended for HCT, and intermediate risk patients were recommended for HCT per local institutional standards.

Our study was limited by its retrospective nature and susceptibility to selection bias. A large majority of patients in our study (88%) had good performance status. Patients with the highest burden of comorbidities from obesity may have been disproportionally excluded due to the strict inclusion criteria. While this limits generalizability of our findings to patients with poor performance status, inclusion of more patients with poor performance status may have introduced more confounding factors in multiple BMI groups. There were also fewer patients with class II obesity than class III obesity in our study, and the sample size of patients with class I and II obesity may have limited our ability to identify a significant effect.

In conclusion, our study found that patients with class III obesity had increased rates of early death, worse OS, and worse OS after transplant as compared to normal weight patients undergoing intensive induction chemotherapy for previously untreated AML. In our cohort, class III obesity portended worse outcomes across multiple time points. Further studies are needed to better characterize patients with AML and class III obesity, and future directions include assessing lean body mass, identifying comorbidities, and prospective studies to assess outcomes. Identifying this at-risk patient population may help guide early weight management interventions for patients with AML.

## Figures and Tables

**Table 1:** Patient characteristics by body mass index (BMI) category. Median (range) or N (%) reported for summary.

	Underweight (n = 9)	Normal weight (n = 182)	Overweight (n = 225)	Class I obesity (n = 145)	Class II obesity (n = 82)	Class III obesity (n = 86)	All	P-value
Randomized arm								
DA	2 (22)	63 (35)	90 (40)	47 (32)	32 (39)	26 (30)	260 (36)	0.13
IA	6 (67)	63 (35)	85 (38)	55 (38)	23 (28)	27 (31)	259 (36)	
IA + Vorinostat	1 (11)	56 (31)	50 (22)	43 (30)	27 (33)	33 (38)	210 (29)	
Age	50 (21,60)	47.5 (19, 60)	51 (19, 60)	51 (18, 60)	47.5 (22, 60)	47 (19, 60)	49 (18, 60)	0.039
Sex								
Female	7 (78)	90 (49)	97 (43)	68 (47)	41 (50)	52 (60)	355 (49)	0.058
Male	2 (22)	92 (51)	128 (57)	77 (53)	41 (50)	34 (40)	374 (51)	
Race								
Asian	1 (11)	6 (3)	8 (4)	1 (1)	0 (0)	1 (1)	17 (2)	0.0075
Black	0 (0)	7 (4)	15 (7)	10 (7)	10 (12)	11 (13)	53 (7)	
Native American or Alaskan	0 (0)	2 (1)	0 (0)	1 (1)	2 (2)	1 (1)	6 (1)	
Native Hawaiian or Pacific Islander	0 (0)	0 (0)	0 (0)	1 (1)	1 (1)	1 (1)	3 (0)	
White	7 (78)	162 (89)	184 (82)	122 (84)	67 (82)	65 (76)	607 (83)	
More than one race	0 (0)	1 (1)	1 (0)	0 (0)	1 (1)	1 (1)	4 (1)	
Unknown race	1 (11)	4 (2)	17 (8)	10 (7)	1 (1)	6 (7)	39 (5)	
Ethnicity								
Hispanic	0 (0)	14 (8)	23 (10)	14 (10)	3 (4)	5 (6)	59 (8)	0.45
Not Hispanic	9 (100)	168 (92)	202 (90)	131 (90)	79 (96)	81 (94)	670 (92)	
Performance status								
ECOG 0–1	9 (100)	169 (93)	198 (88)	128 (88)	71 (87)	63 (74)	638 (88)	0.0015
ECOG 2–3	0 (0)	13 (7)	27 (12)	17 (12)	11 (13)	22 (26)	90 (12)	
Ontology								
De novo	8 (89)	162 (89)	208 (92)	129 (89)	74 (90)	76 (88)	657 (90)	0.73
Secondary	1 (11)	20 (11)	17 (8)	16 (11)	8 (10)	10 (12)	72 (10)	
Cytogenetic risk								
Low risk	0 (0)	23 (13)	20 (9)	26 (18)	13 (16)	13 (15)	95 (13)	0.085
Intermediate risk	7 (78)	110 (60)	147 (65)	92 (64)	46 (56)	56 (65)	458 (63)	
Adverse risk	2 (22)	46 (25)	53 (24)	20 (14)	22 (27)	13 (15)	156 (21)	
Unknown risk	0 (0)	3 (2)	4 (2)	6 (4)	1 (1)	4 (5)	18 (2)	

Abbreviations: daunorubicin and cytarabine (DA); idarubicin and cytarabine (IA); Eastern Cooperative Oncology Group (ECOG).

## Data Availability

The dataset analyzed for this study will be available through the NCTN data archive: https://nctn-data-archive.nci.nih.gov.
